# When hospitals came to Sweden in the eighteenth century: a foreign import with practical difficulties

**DOI:** 10.1017/mdh.2025.10030

**Published:** 2026-01

**Authors:** Maria Sjöberg

**Affiliations:** Department of Historical Studies, University of Gothenburg, Gothenburg, Sweden

**Keywords:** Eighteenth-century hospital, Justification, Economy, Military, Ecclesiastical responsibility, Path dependency

## Abstract

Since Foucault’s seminal work in the 1960s on the consequences of eighteenth-century discursive shifts in medicine, the establishment of hospitals during this period has often been interpreted as a progressive innovation driven primarily by medical scientists. However, less attention has been given to the ways in which the founding of hospitals was intertwined with domestic traditions and the practical challenges inherent in their implementation. By examining the establishment of the Seraphim Hospital in Stockholm, along with subsequent hospital foundations in Sweden, the practical difficulties involved become evident. Some of these challenges, particularly those related to funding difficulties, bear a striking resemblance to contemporary discussions on enhancing the efficiency of healthcare, despite the differing historical contexts. In the Swedish eighteenth-century context, ecclesiastical authority in medical matters persisted and played a role in the establishment process, while the military character of the kingdom also influenced hospital development. The conclusion drawn is that both national and local conditions shaped how medical reforms were conceived and practised. The historiographical emphasis on novelty and change may, at times, obscure the continuity of past practices, which undeniably played a crucial role in shaping the new. The concept of path dependency is thus employed not only to trace these historical connections but also to explore the ways in which they influenced the Swedish context, ultimately shaping the trajectory of hospital development in the country.

## Introduction

The eighteenth century saw an expansion of hospital establishments in Europe.^1^ Among others, the Charité in Berlin was built in the 1710s.^2^ In London, Guy’s Hospital, St George’s Hospital, the Middlesex Hospital and the London Infirmary, later the Royal London Hospital, opened.^3^ In Copenhagen, Kongelige Fredriks Hospital and Almindeligt Hospital were established.^4^ In Stavanger, a hospital was established in 1773.^5^ In Paris, Hôpital Necker and Hôpital Beaujon complemented the ancient Hôtel Dieu. The Royal Infirmary of Edinburgh was established in 1729, and in Vienna, the Allgemeines Krankenhaus opened in 1784.^6^ In Sweden, the Seraphim Hospital (Sw. Serafimerlasarettet) was established.^7^ The list of new hospitals in Europe could be longer. Prior to the European establishment of hospitals in the eighteenth century, the institution had a long and diverse history, tracing its origins from Byzantium through Islamic cultures to Asia, and eventually to Europe, where medieval hospitals were closely associated with monasticism.^8^ Thus, the establishment of hospitals in Europe during the eighteenth century can be characterised as a second or third wave movement. What they all had in common was that they were intended to cure the sick and poor while promoting education and science. This initiated a change whereby the sick would no longer be cured in their homes or a church medical institution. In the long term, a division between the spiritual and the physical took place. The church and the clergy, who had previously managed hospitals, and also acted as practitioners of medicine in their respective congregations, were pushed away from the bodily-orientated medical care, which was increasingly transferred to medical expertise, the doctors.

The Seraphim Hospital opened in 1752, and in the 1760s, several hospitals were established around the country. In addition to providing medical care for the sick, Seraphim Hospital facilitated scientific research, and internships at the institution soon became a requisite component of medical training. The two institutions that preceded Seraphim Hospital served distinct purposes. Danviken Hospital, established in the sixteenth century, functioned as a refuge for the impoverished, infirm, and ill, whereas Uppsala University Hospital, founded in 1708, operated as a modest teaching hospital. Individuals in need of medical care were therefore referred to a limited number of private practitioners, while in rural areas, care was primarily provided by lay healers, including priests, wise women, and elderly men. Hospitals established after Seraphim Hospital, so-called county hospitals, were exclusively devoted to the care of patients. The sudden founding of hospitals raises several questions. How were they justified, and how were the necessary resources mobilised? The following section, therefore, examines the arguments of the proponents as well as the financial solutions adopted for the Seraphim Hospital. At the same time, and more importantly, an insight is provided into what kind of institution an eighteenth-century hospital in Sweden actually was. Table 1 shows the establishment of hospitals in Sweden in figures:

**Table 1.** Hospitals established in Sweden

Time	Number
1750–1781	18
1782–1811	20

Note: Wawrinsky’s compilation is not complete. It overlooks the smaller hospitals initiated by mills and other private companies, and also the hospitals in Finland. Several of the smaller hospitals operated only for a short period, which complicates the picture. In general, it is reasonable to assume that the numbers in Table 1 represent a minimum.

Source: Richard Wawrinsky, *Sveriges lasarettsväsende förr och nu: ett stycke svensk kulturhistoria*, (Stockholm: author’s publisher 1906), appendix 1.

From a starting point close to zero, almost forty hospitals were established in just over half a century. The growth was admittedly not quite as dramatic if the number of beds is added to the figures in Table 1. In 1781, there were a total of 196 beds at all eighteen hospitals in the country, and in 1811, the figure was 692.^9^ If these figures are compared with the situation at the turn of the nineteenth century, when the number of hospitals was seventy-six and the number of beds 7,353, it is clear that the major breakthrough for an expanding institutionalised healthcare system did not take place until later, in the nineteenth century. However, the development began in the eighteenth century, and the figures confirm that this was a time of change, a turning point.

What made this era particularly amenable to healthcare in specialised institutions? Foucault’s answer, which specifically links the changing role of medical science to the authorities’ need to control epidemics, points to the discursively mutually supportive relationship between the state and doctors, while the medical historian Guenter B. Risse underscores the significance of philanthropic and religious motives and professional interests within the British context.^10^ John Henderson, Peregrine Horden, and Alessandro Pastore, akin to Risse, underscore that hospitals have held distinct meanings across various times and locations, contending that the process of medicalisation is more complex than Michel Foucault’s perspective implies. Simultaneously, Henderson et al observe that ‘[Foucault´s] identification of the French Revolution as the brief but explosive period of ‘the birth of the clinic’ has proved so influential that historians have to continue rehearsing it even though few of them now accept it’.^11^

Were hospitals an obvious response to the problems to be solved, however? In his study of how German doctors debated healthcare in the late eighteenth century, medical historian Fritz Dross, with a critical eye on Foucault, points out that modern hospitals fit remarkably well into the medical discourse of the late eighteenth century. The doctors in Dross’s study who advocated hospitals claimed better medical care and efficiency gains by having more patients ready for treatment in the same place. At the same time, other doctors also invoked quality and, therefore, believed that the sick should be cared for at home. In retrospect, the pro-hospital doctors appear to have been dominant, and hospitals were the obvious choice, despite the fact that several hospital projects met with resistance and were therefore never realised.^12^ Dross’s study shows that although the hospital issue was driven by doctors, the public sector, authorised by political leaders, was responsible for financing the hospitals.^13^

In Great Britain, where extensive historical research is being conducted on the organisational changes in hospital care, it appears that eighteenth-century hospitals were generally initiated and financed by donations from a wealthy local elite and that parts of the hospital work were voluntary.^14^ Gift-giving has a long history and was recognised as an important gesture of generosity as well as a point of departure for a mutual exchange transaction in the future.^15^ Gifts built bonds of loyalty but were also part of a power-building process. In the medieval princely ideal, for instance, the prince would be generous with gifts as an aspect of his exercise of power.^16^ The tradition of the rich giving their abundance to the needy was clearly strong even in social classes that were not considered princely. In Great Britain, local and financially strong elites were the driving force, not the king, doctors or state authorities.^17^ A similar pattern, in which private charity enabled the establishment of hospitals, emerged in several European countries, including Germany and Italy.^18^ This pattern varied. In Austria, Joseph II was a direct driving force behind the establishment of the Allgemeines Krankenhaus.^19^ In France, hospitals had long been an integral part of the church organisation. During the seventeenth century, royal political control increased. However, Tim McHugh’s study shows that in practice, royal regulations did not play much of a role. Local social elites were responsible for funding both hospitals and poor relief.^20^ The royal attempts to control and organise from a central point were thus mainly surface phenomena.

In Norway, as in most countries, the responsibility and costs shifted over time between the church, local communities, and the state, but there were also collaborations. For example, in the establishment of the first hospital in Northern Norway in 1796, the aim of which was to control venereal disease, the Danish authorities were the driving force, but local support was provided by the local clergyman, who, like several other clergymen, also still practised medicine himself.^21^ The Norwegian historian Ole Georg Moseng, who has investigated the matter more closely, believes that after 1750 a change in attitude took place. In the new belief that it was possible to make sick people healthy, many measures were taken, including the appointment of district doctors and the founding of hospitals.^22^

Regardless of what the driving forces were, the establishment of the eighteenth-century hospital is in general interpreted as something qualitatively new. In Guenter B. Risse’s typology, the eighteenth-century hospital was oriented around the provision of care, in contrast to the medieval hospital, which was focused on the concept of mercy. In both instances, the hospital served as a symbolic reflection of the prevailing social and ideological values of the time.^23^ However, it may be noted that many of the hospitals established in various places in the eighteenth century were based on a reorganisation of the older hospitals. The new change was based on lessons learnt from the old. In sociological and political science research, studying macro-processes and changing policies, the concept of path dependency is used to capture continuity, while a change, or a historical event, in which multiple alternatives are possible, is referred to as a critical juncture.^24^ As the sociologist Walter Korpi emphasises in his studies of the emergence of welfare institutions in the twentieth century, the old, or path dependency, provides an explanatory context for regional and national differences in the way organisations were designed and financed.^25^

The conceptual pair, path dependency and critical juncture, has its own history and is difficult to define in absolute terms, which also causes confusion.^26^ However, the sociologist James Mahoney’s definition that ‘path dependence characterises specifically those historical sequences in which contingent events set into motion institutional patterns or event chains that have deterministic properties’ is widely spread in macro level studies and could, perhaps, be useful on an overall level in this case as well.^27^ Here, the conceptual pair path dependency and critical juncture are guiding a micro level analysis in order to clarify how hospital establishment in eighteenth-century Sweden was carried out in practice. They simply help to study the establishment of hospitals from a historical perspective, i.e. from the experience of the eighteenth century. The hospitals of that time were not the same as those of a century later, nor should they be studied as precursors to them.^28^ This is in line with current research on the history of these institutions, in which the emphasis is on the complications of issues surrounding responsibility, organisational design and financing, not least because the answer shows whether healthcare was considered a right as in the present, or a gift as in the past.^29^ In eighteenth-century Denmark, to which Norway belonged until 1814, the establishment of hospitals can also be linked to a coexisting, simultaneous ideological shift: the promotion of population growth and work capacity.^30^ The same was true in Sweden. With a healthy and able-bodied population, the poor and barren country in the north could be transformed into a prosperous one. Therefore, the poor and sick had to be given adequate care so that they could promptly be set to work. Historian Karin Johannisson articulates the vision of the time: ‘With the help of myriads of hard-working labourers, Sweden would be transformed from a “cold, wild desert” into a land of teeming prosperity’.^31^

Based on the optimistic visions of the time, government authorities in Sweden should have been as active as those in Denmark and Norway in establishing a capable hospital organisation. Older historical research carried out by researchers who were doctors themselves consequently emphasises the efforts made jointly by the authorities and the representatives of medical expertise.^32^ Long-term factors are also emphasised. Physician and medical historian Otto E. A. Hjelt’s extensive three-volume work on the development of Swedish and Finnish medical care emphasises the importance of education. As long as interest in domestic medical education was weak, both science and clinical activities suffered. However, the establishment of the Collegium Medicum in 1663 helped to strengthen education and regulate the practice of medicine.^33^ This paved the way for hospitals and medical centres. Physician and medical historian Richard Wawrinsky, who has studied the organisational aspects of the emergence of the hospital system, stresses the importance of the care provided by the state authorities. Like an old-fashioned patriarch, they provided the hospital with funds for maintenance and exercised ‘benevolent control’ over both enterprises and improvements designed to develop and expand the hospital.^34^ Physician and medical historian Wolfram Kock, who studied the chronological development of the Seraphim Hospital from its inception until 1952, emphasises that physical healthcare had also been discussed in the past, but the needs had not been met. Before the eighteenth century, the people and the political leaders were too preoccupied with foreign policy and did not have the strength to address social issues seriously. Kock also argues that there was a need for a ‘scientific maturity’ that was previously lacking. ‘Men like Urban Hjärne, Olof Rudbeck, Johan von Hoorn, Carl von Linné, and Nils Rosén von Rosenstein’ contributed to this.^35^ Referring to the Swedish war losses during the Great Northern War, and the impoverishment, poverty and disease that followed, Wolfram Kock characterises Sweden’s eighteenth century ‘as a very sick century’.^36^

Patriarchal care, the long-term reinforcement of education, the clinical picture and scientific maturity summarise the explanations given by older Swedish research for the establishment of hospitals in the eighteenth century. The century is presented as a watershed in relation to previous centuries, and political leaders and medical expertise are emphasised as the driving forces on the path from darkness to light. In retrospect, these interpretations, which were formulated during the first fifty years or so of the twentieth century, can be reconciled with Foucault’s perspective by the fact that some of the foremost representatives of medicine succeeded in getting the need for hospitals accepted by the authorities, the Diet (Sw. Riksdag) and the king, but they can also be seen as an uncritical success story in which the social pathos of medical science, combined with the benevolence of the authorities, plays a key role.^37^ In Swedish research, this view has not been subjected to fundamental scrutiny, partly due to the general shift in the history of medicine towards other questions in the history of ideas.^38^

In Sweden, the Church Act of 1686 stipulated that church officials were responsible for the healthcare of their subjects.^39^ However, the Church Act did not stipulate how medical care should be practised, financed, or organised. Through the documentation of the creation of the Seraphim Hospital, it is possible to delve more deeply into both the question of responsibility and how the practical work was intended to function. In this way, more insight can be gained into what kind of functions an eighteenth-century hospital in Sweden would fulfil. Since hospital archives are generally thinning out and parts of documents have thus been lost, the rich documentation in older literature is used here to a large extent as a basis.^40^

## A hospital for Stockholm’s soldiers and the development of science

The assessor of the Collegium Medicum, Nils Boy, who was also the city physician in Stockholm, submitted memoranda to the Diet in 1731 and 1734 on the importance of a hospital in the capital. Foreign countries were a model. According to Boy, the hospital was needed for poor and sick people as well as for medical education. The magistrate in Stockholm was asked to comment, and it appears that the magistrate was certainly aware of foreign institutions that were paid for with public funds. Unfortunately, as the city lacked financial resources and also had other public buildings to consider, the proposal could not be supported. Instead, reference was made to Danviken Hospital, which offered care for the poor and sick in the summer.^41^

Financial constraints stopped Nils Boy’s proposal, but the issue was taken forward by the Health Commission set up in 1737. The Commission’s letter mentions the benefits of hospitals abroad and that Stockholm should follow suit. However, the hospital should not only serve the city but the whole country. This would limit the spread of infectious diseases. Otherwise, the risk was that the infections of the poor would affect many more people, with the imminent danger of entire cities going under. Another reason was to prevent the premature death of the poor, partly because they were exposed to harmful treatment by ignorant people around the country. Even though the ignorant knew nothing about disease or drugs, they offered cures that were, in fact, life-threatening. A successful health centre, where patients were cured, could compete with such treatments. In the long term, the population would grow, which was in line with the mercantilist ideas of the time. Moreover, a domestic hospital could utilise herbs and crops that grew within the country’s borders. If it turned out that domestic plants could replace foreign ones, future savings would be substantial. However, knowledge of the medical effects was considered to be scanty and needed to be elaborated. The exploration could be done with ‘careful’ experiments on the sick, ‘for such experiments cannot be employed more comfortably than in hospitals and public asylums’. Such trials were easier to carry out under constant supervision, which was not possible if the sick were treated in their private homes. Another motive was that young medical students returning from their educational trips needed practical training under the guidance of experienced doctors. The hospital would provide this, and practical exercises were seen as a more effective way of learning than ‘many years of tedious reading at the academies’.^42^ The hospital also had the possibility of conducting postmortems on the bodies of those who had died there. This would make it easier to determine the cause of death and further increase knowledge of the nature of diseases.

The response was positive. Only one stumbling block remained, although it was a crucial one, namely, funding. The government (Sw. Riksrådet) entrusted one of the many deputations of the ‘age of liberty’ (1718–72) to investigate funding. The financing proposal was based on eight sources of funding. All were fees, taxes or donations, but the poor were to be guaranteed free healthcare, as a gift. Registers of voluntary donations handled by the church would be set up in both urban and rural areas. Taxes were to be levied every time the ‘Swedish Comedy Theatre’ performed, with double taxes for foreign performances. Incoming and outgoing ships had to pay taxes in relation to their cargo, and the same applied to bringing hay into the city. The granting of privileges and transport was to be subject to charges. Anatomical demonstrations on the bodies of the deceased could be made for a fee, and those who wanted to learn about surgery and medicine by visiting the hospital would also have to pay for themselves. It was also proposed that patients who were not poor should be charged for medical care.^43^

When the issue of the hospital, which had hitherto been initiated by the representatives of medicine, had reached this stage, it was taken further by the military. At the parliamentary meeting of 1738–39, Lieutenant Stierneld of the nobility submitted a proposal calling for the establishment of a hospital for the city’s sick, in particular wounded soldiers and the poor. City officials were also to be offered care. The argumentation followed that of the Commission, including the comparison with other countries, in which Swedish conditions were presented as particularly backwards. Like other countries, Sweden also needed a hospital. Stierneld also proposed a general and concrete solution for financing, namely the organisation of a lottery from which twelve per cent of the proceeds would go to the foundation of the hospital. The proposal was supported by the Secretarial Committee (Sw. Sekreta utskottet) and, together with the deputation’s proposal, it now became a matter for the four estates in the Diet to decide.^44^

The knights’ and nobility’s estate approved the deputation’s proposal and had no objections to Stierneld’s proposal either. The other three estates said no to the deputation’s proposal but yes to Stierneld’s suggestion. All in all, the estates’ decision meant that the deputation’s proposal was rejected while Stierneld’s was supported by all. There was thus no longer any question of offering the entire country’s inhabitants medical care at the hospital. Only the city’s soldiers and the poor were included.^45^

In the spring of 1739, the King confirmed that the future hospital would be established to serve the poor and the two regiments located in Stockholm. At the same time, it would also be an educational institution. Financing would be as proposed by the deputation, but without the fees and taxes that the peasantry and bourgeoisie had opposed. In addition, a special lottery was to be organised in accordance with Lieutenant Stierneld’s proposal. The Diet of 1741 approved the decision to build a hospital in Stockholm.^46^ Now all that remained was to realise the plans.

## The entire country contributed

In his work on the history of the Swedish and Finnish medical services, the above-mentioned Otto Hjelt gives a detailed account of how the fundraising was carried out. It shows that collection was slow; several encouraging ordinances were issued, and the collection of fees was extended to more areas than those in both Stierneld’s proposal and the deputation’s report. Charges were levied on the awarding of journeyman’s and master’s certificates, as well as on promotions and letters of privilege. These fees mainly affected people in the cities, especially those who lived in Stockholm. In addition, it was decided that gifts should be collected at christenings and weddings. Parish bell-ringers were instructed to go around among the guests, collecting gifts on a plate. Godparents were encouraged to contribute, but the collection went poorly. After the collection was reluctantly taken over by the clergy, it increased slightly. Additional funds came from paid entertainment. A levy on each deck of cards sold was introduced, as were fees for performances at the Ball House and for running Stockholm’s four billiard halls. The collections in all the country’s churches on the first Sunday of Advent went to the Seraphim Hospital. Later, they came to belong to the county hospitals, and this fundraising did not cease until 1887.^47^

The authorities’ contribution consisted of administering the levies collected, but no tangible financial contribution of their own. It was not until 1783 that some of the fees were transformed into an annual appropriation.^48^ For several decades, the clergy in the parishes throughout the country played a key role. The fund set up to administer the resources of the hospital (Sw. Lasarettsfonden) was reinforced with funds stemming from the regulation that every person leaving the countryside to move to the cities had to pay a fee before a certificate of removal could be issued by the local clergyman.^49^ It was thus up to the clergy to collect the transfer fee. If several fees affected the inhabitants of cities more than the countryside, especially the inhabitants of Stockholm, the transfer fee was of the opposite nature, and it also caused difficulties of interpretation for the various consistories around the country.^50^ However, the largest income to the hospital was the lottery proposed by Stierneld. Until 1805, the lottery provided a significant annual income for the hospital.^51^

In terms of responsibility as expressed in the economic conditions, both church and civilian officials were involved in the fundraising efforts. However, the funds raised came from the population around the country. The combination of fees, taxes and donations meant that the economy of the Seraphim Hospital followed what seems to have been the usual pattern for both eighteenth-century and later European hospital foundations, for which the authorities’ support required private funds.^52^ The hospital was established in a ‘mixed welfare economy’ in which private charity of various kinds played a key role.^53^ Some of the private funds that went to the Seraphim Hospital were not included in the original calculation, namely, voluntary donations. In his history of the Seraphim Hospital, Wolfram Kock provides a list of donations to the hospital from the 1750s to the twentieth century.^54^ They were of varying sizes, given by women and men, and in many cases subject to testamentary conditions. The gifts provide a glimpse of the hospital’s colourful history. During the first decades of the twentieth century, for example, several donations were made to purchase instruments for the hospital’s X-ray department. However, donors in the nineteenth century were the most numerous, and their social origins also varied the most. Clergymen, doctors, matrons, merchants, and highly ranked government officials are listed as donors of varying sums, and many were specifically intended for the care of the sick and poor. Among the eighteenth-century donations, of which the donors belonged to the upper echelons of society, the gift that the hospital received from the city’s bourgeoisie on the occasion of Gustav III’s recovery from a broken arm is notable. A gift was to cover the maintenance of four hospital beds for the care of arm and leg fractures. The gift of the Russian Empress Catherine II was unconditional, while the Austrian diplomat Theodor Christoffer von Antivaris stipulated that his gift, intended for the maintenance of four hospital beds in two rooms, one room for women and one for men, was destined for Roman Catholics only.

All in all, it was on the basis of numerous fees and donations and a lottery that the hospital was planned. The church’s role in fundraising confirms that social issues, medical care, and care for the poor were part of its overall responsibility, but despite that, not everyone had that right. Target groups were only soldiers and the poor in Stockholm. Nonetheless, the money-raising involved the entire kingdom, even though the hospital only intended to treat inhabitants of Stockholm. The duty of issuing certification of the need for medical care fell to the clergy and not the medical scientists. The links to the past, path dependency, may be clarified in two main ways. The role of the clergy and the church, both in fundraising and in acting as the first line of healthcare, is reminiscent of the role of the church before the sixteenth-century Protestant Reformation. Similarly, the contribution of the population in the form of collections and special gifts for the care of the poor was a traditional source of funding. The role of the clergy was reinforced in terms of financial management but diminished in their involvement in medical activities. Nevertheless, through their authority to issue certifications, the clergy retained a central role as the primary arbiters in determining eligibility for medical care.

## The doctor’s speech and the barber-surgeon’s document

More than a decade after the first proposals on the subject were submitted, in the mid-1740s, the hospital was still only at the planning stage. In 1746, the subject was raised again in the Royal Swedish Academy of Sciences. Abraham Bäck, president of the Collegium Medicum, gave a speech arguing in favour of a hospital in Stockholm.^55^ Medical science needed knowledge about both sick and healthy bodies. This required a centralised and controlled supply of patients, which was not possible to achieve in the homes of the sick. However, Bäck believed that the general public would also benefit from a hospital. In the event of plagues in the country, the necessary doctors, trained in the hospital, could be sent to the places where they were most needed. Another benefit was the ability to cure. The conditions for the soldiers whom the barber surgeons in Stockholm were now visiting ‘in a small, leaky alcove, in poverty and misery, without anyone taking proper care of their food and drink or taking proper care of the necessary medical treatment’ were, according to Bäck, unsatisfactory.^56^

Like his predecessors, Bäck emphasised that conditions in Sweden were particularly poor. Provincial doctors were too few, and the common people’s home remedies were inadequate. Even in Russia, there were now hospitals, and Bäck asked himself: ‘Who would have thought that we would ever have to learn from Russia?’.^57^ Bäck listed several hospitals in other countries, where medical care was provided by doctors in collaboration with barber surgeons, and he gave a detailed account of their organisation and financing. Voluntary gifts were emphasised as both fundamental and exemplary. The main French hospital, Hôtel Dieu in Paris, was also the largest. When Bäck himself visited the hospital, the number of patients and staff totalled 4,000. However, there was no need to think about such a large number in Sweden at a time when financial resources were scarce. Bäck cited several foreign examples of care institutions that had started on a small scale and then become large and reputable.^58^

The same year that Bäck gave his speech, another member of the Royal Swedish Academy of Sciences, Olof Acrel, published a document on the same subject. Like Bäck, Acrel cited foreign experience. He had previously served as a barber surgeon (Sw. fältskär) in the French army. He had managed a large hospital there and acquired the expertise required to design a hospital that would have the capacity for a relatively large number of patients.^59^ Like Bäck, Acrel also cited the benefits of a hospital. As his first point, he pointed to the many sick people in need, ‘who in misery perish, and for lack of proper care, are treated here, cured and sent back to their work healthy’. Acrel’s second point meant that not only soldiers and the poor in Stockholm would benefit from the hospital: ‘That those in the countryside who are helpless when severe injuries have occurred, can apply here, be admitted and, as far as possible, be cured without compensation’.^60^

Concern for the misery of the poor was present in both Bäck’s and Acrel’s arguments. At the same time, the value of making the sick healthy so that they could return to work as soon as possible was emphasised. In both texts, the greatest emphasis was placed on how a hospital could be realised in practice and be of socio-political benefit. From initially being a nationwide hospital to later being limited to only the city’s soldiers and poor, Acrel nevertheless assumed that the seriously ill and injured in the countryside would also be able to use the hospital, though not unconditionally. The hospital was intended to cure the poor of curable diseases. Unlike in older hospitals, there was no room for the incurable in either Bäck’s or Acrel’s arguments.

Two years after Bäck’s speech and Acrel’s document, in 1748, the Order of the Seraphim was established, which later became the name of the hospital. According to the statutes, the order’s eight knights had the privilege of being responsible for the welfare of the sick and the poor.^61^ Abraham Bäck was hired for the concrete planning. Two Seraphim knights were placed on the board of directors, who later came to run the hospital.^62^

The hospital opened on 30 October 1752. At that time, there were only eight beds in two rooms; six months later, there were eighteen beds in four rooms, with men and women separately. A very limited number of sick people could thus be admitted for treatment.^63^ It goes without saying that all needs for care among the nation’s poor were not met by the Seraphim Hospital. Not even those living in Stockholm could count on a bed. The Seraphim Hospital was very small. By way of comparison, the Kongelige Fredriks Hospital in Copenhagen opened in 1756 with 300 beds, while the Middlesex Hospital in London, which opened in 1748, had 30 beds, which, however, increased to 55 in the mid-1750s.^64^ The Edinburgh Infirmary opened in 1729 with room for 35 patients, while the Allgemeines Krankenhaus in Vienna opened its doors in 1784 and was designed for 2,000 patients.^65^

Despite its small size, the Seraphim Hospital, like the hospitals abroad, was organised into two departments. Until 1753, Abraham Bäck was the hospital’s chief physician in charge of internal medicine, and until the turn of the century, 1806, Olof Acrel was its chief barber surgeon in charge of surgery. Both Abraham Bäck and Olof Acrel kept a kind of diary of their clinical activities.^66^ Acrel also collected his experiences and published a selection of them as case histories in two editions.^67^ Two decades of discussion about the hospital’s creation, in which these two were the driving force, were over. The economy, from having been a stumbling block, had now made the hospital possible through the lottery and the public’s fees, taxes and donations. However, the discussion was not over. The direction, financing, organisation and activities of the hospital were still being questioned.^68^

## Inside the hospital

The rules for the hospital, issued by the hospital board in 1755, set out what both staff and patients had to abide by. Piety was the foundation of the institution. Every Sunday and public holiday, therefore, church services were held in the hospital. Morning and evening prayers were said by the doctor or one of the patients.^69^ Those seeking care were required to obtain an attestation from a clergyman or master. At the time of enrolment, the patient’s own clothes were replaced by those of the hospital. No food or alcohol was allowed, and smoking was forbidden. Patients were not allowed outdoors during their stay in the hospital and were forbidden to accept or beg for money from strangers. Anyone who violated these rules lost their place in the hospital. The doctor and the chief barber surgeon were responsible for enrolment and had to visit the sick at least once a day and keep a diary. The food regime for the sick was fixed and strictly budgeted. Births were attended by the doctor or a trained midwife. Both the doctor and the barber-surgeon had to teach students in their respective fields. The hospital’s pharmacist was only allowed to mix medicine according to the prescriptions of the barber-surgeon and the doctor. Finances were the responsibility of the manager, who had to ensure that the farmhands and orderlies took care of the equipment and left the food untouched for the sick. In addition to outdoor work, the farmhands, like the orderlies, had to care for the sick. Both were to ‘show the sick all courtesy and tenderness, and never treat them with harsh words’.^70^ In addition, the orderlies were responsible for cleaning, sweeping and airing the sickrooms, making beds and scattering chopped spruce wood on the floors. Both farmhands and orderlies were expected to live in the hospital to be constantly available to the sick: ‘These servants should always be present in their rooms, so that if any of the sick require it, they can assist them’.^71^

Staff and patients were kept on a tight rein. With public access, the hospital was still an open and outward-looking institution. Visitors were free to visit the sick, witness surgeries and other activities in the hospital. Summarised annual reports were also published in the daily press, describing the activities, the number of patients treated, their diagnoses and the procedures carried out, etc.^72^ In 1753, practical training in the hospital became a compulsory part of medical training. The unpaid trainees had to participate in the work of the hospital in the same way as the doctor and the barber-surgeon.

The hierarchy within the staff was clear. The scientifically trained but unpaid staff, the doctor and the barber surgeon, were superior to the care-orientated staff, the orderlies and the farmhands. Since poverty was a criterion for admission to the hospital, the social status of the patients was at about the same low level. A list of the first patients treated by Abraham Bäck can be found in preserved hospital records, reproduced in facsimile by Wolfram Kock. It shows that a total of fifteen patients received medical care, four women and eleven men, and that various types of chills and fevers predominated.^73^ The men’s occupations were simple crafts, and only one was a soldier (artilleryman Brandbom). The women’s occupations were not prestigious either; two were listed as ‘poor maid’, one was listed as ‘pregnant woman’, and the fourth was listed as the wife of an artilleryman. In addition, a handwritten memorandum in 1752 states that the unmarried Kerstin, thirty years old and four months pregnant, was being treated for jaundice in the maternity ward.^74^ Considering the nature of the diseases, fever and the like, the preponderance of male patients has no immediate explanation. The men, like some of the women, were poor and unmarried. For both women and men, proper nutrition and rest were probably the most effective cures that eighteenth-century medicine could offer.

The medical experts’ view of what the hospital required was unequivocal. Both lack of rooms and lack of funds, and surprisingly, also lack of patients were emphasised. Johan Lorenz Odhelius, who was the third physician to succeed Abraham Bäck, served from 1772 to 1813 and, like his colleague Acrel, had a military background. In a speech to the Royal Swedish Academy of Sciences in 1776, he outlined the history of the hospital, pointing out that since 1752, when ‘15 miserable wretches’ had been cared for and helped during the remaining months of the year, only one of them had died.^75^ The following year, the number of patients was ninety-three, of whom eighty-three were from Stockholm, the rest from the countryside. Five died. Odhelius’ speech includes a review of each year’s number of patients and births from 1752 to 1776. During these years, the hospital received a total of 8,261 patients, 6,425 from Stockholm and 1,836 from other places, reasonably close to Stockholm. During the same period, 369 children were born at the hospital. Of the sick, 927 died.

Odhelius explained the high death rate: ‘The general temperament of our people, who never seek help sooner than extreme necessity requires, but suffer more than anything else, for as long as possible, and let the Quacks deceive them right down to their clothes’.^76^ He also pointed out that if the hospital had more beds, more people could be helped while they still had the strength to live. Now, ‘dying persons are often thrown into the yard, who must then be admitted, and only increase the death list and the cost of burial. It has also happened that dead people have been taken there and thrown in’.^77^ Odhelius believed that both the sick and science would benefit from both doctors living in the hospital, but then their fees would have to be increased so that they would not have to take on the care of the sick ‘outside the building’ for their livelihood.^78^

The doctors’ work was poorly remunerated; initially, they were not paid a salary at all. Their respective private practices were their main source of income. Towards the end of his long speech, Odhelius returned to the need for more beds in the hospital, but he said that a hundred would be enough. The reason he gave was that people distrusted public hospitals and were reluctant to go there, and that the risk of infection was less in Sweden than in other countries.^79^ There would therefore be no need for more than a hundred beds. Another reason, not mentioned by Odhelius, was that at the time of his speech, in 1776, the Seraphim Hospital was no longer the only hospital in the country. County hospitals had now been established in several places, and a significant background was the questionable economic conditions at the Seraphim Hospital.

## Discussions about costs and the actors: the deputation

The existence of the hospital after 1752 could not be taken for granted. Various localities outside the vicinity of Stockholm requested exemption from the obligation to send funds for its maintenance. As a rule, such requests were rejected except for Finland. The funds collected there were specially aimed at founding the Turku hospital, which was established in 1759. Criticism of the management of the Seraphim Hospital and the excessive costs incurred by the entire kingdom was voiced ever more frequently and loudly, and eventually became a matter of parliamentary concern. The clergy, who played an important role in raising funds, appointed one of the church leaders, Jacob Serenius, to examine the hospital closely. His letter, which was favourable to the hospital as such, deeply regretted that the hospital system had started so late in Sweden, and that other countries had a hundred-year head start. At the same time, he noted that for the same amount of money that was spent on a hospital in Sweden, a hospital in England could finance twice as much, and with better facilities. Until an inquiry was set up, the ecclesiastical dioceses around the country must be allowed to run their own hospitals; preparations had already been made in his own diocese, Strängnäs. This would make healthcare more accessible in rural areas and relieve the burden on the Seraphim Hospital. As a result, the counties would be able to avoid the costs of the Serafim Hospital.^80^

This criticism resulted in a deputation (Sw. Lasarettsdeputationen) which was assigned to investigate the management of the hospital. It consisted of representatives of all estates in the Diet. The deputation’s examination enables further insight into the hospital’s activities while also highlighting contemporary criticism.

The eighteen questions that the hospital deputation sought answers to were very detailed, ranging from meal and staff costs and how effectively the property was used to how the estates of deceased patients were handled. Everything was about finances, nothing about the medical care provided. The questions show that the hospital was supposed to take in sick people from the countryside, but that this was not done as much as the critics wanted. The terms of the donation that the deputation enquired about concerned only the Austrian envoy’s donation, where the testator’s funds were intended for the care of Catholics in special rooms. There were doubts that this was actually fulfilled. Answers to all questions were required from the chief physician Darelius, who had succeeded Bäck, the chief barber surgeon Acrel and the director Kirstein, who all defended the functioning of the hospital.^81^ The purely economic questions were mainly left for Kirstein, while the medical questions were answered by both doctors. The questions and answers provided give further insights into what kind of institution an eighteenth-century hospital really was.

When asked what space was used to accommodate sick people from the countryside while waiting for a free bed, Olof Acrel replied that there was no such room and that it would be detrimental to the hospital. It was better to use the few beds to care for the sick. The deputation also wanted to economise on food. It should be adapted to the number of healthy and sick people, so that the portions that the sick could not eat could be saved. Acrel pointed out that the food programme was hired out to a marketer at a fixed price. The food that might be left over was his salary benefit.^82^ The deputation also wanted to know the principles by which the hospital admitted the sick. Acrel explained the procedure. Patients were admitted based on certificates issued by the clergy of local parishes. For factory workers, the supervisory authority (Sw. Hallrätten) issued certificates. Some crafts offices paid for the medical care of journeymen, and it was also possible for individual heads of households to obtain medical care for their servants at a cost. Acrel pointed out that he had, from the outset, equipped the hospital with his own instruments, carried out experiments in the hospital at his own expense and paid for the publication of the work *Chirurgiske händelser (Surgical Events)* with his own funds, without expecting any reward other than the continued existence of the hospital. Finally, he criticised those who ‘[…] spread demonstrable untruths to the public. I, however, regard it with contempt and am sure of better judgement from those who worship fairness and truth’.^83^

Darelius’ answer was in line with Acrel’s, but he also added an explanation on how the donation for Catholics was used. Only if a certificate could prove their Catholic faith would special rooms be available. However, he also pointed out that several farmers, who had organised fundraising for the hospital, now demanded that their servants should be freely granted medical care by the hospital, which was not possible due to the constant lack of space. He also wrote that many sick people were left outside the gate or in the courtyard in such a miserable condition that they died after only a few hours. No wonder there was dissatisfaction. Darelius emphasised the underestimation of dimensions and argued that the hospital could not handle plagues on an estate, in a parish, or in a factory. There were simply too few beds.^84^ In order to help the sick, Darelius had set up a private clinic, giving advice and medication to those who had not been admitted to the hospital at no cost to the patient. In the past two years, he had treated 700 patients in this way.

Both Darelius and Acrel emphasised private sacrifices for the sake of the hospital. The director Kirstein made no such claim. In response to the question of measures taken to ensure that the sick were not crowded, Kirstein stated that now that they had ten rooms with forty beds, there was no longer any crowding. But he also pointed out that if the hospital’s finances were stronger, another thirty beds could be provided. Regarding staff costs, Kirstein pointed out that he believed that the hospital’s salaries were the lowest that could be paid. He himself and the hospital clergyman had no salary ‘on budget’. Personnel costs included only the accommodation of one journeyman, two farmhands’ wages and half board, three orderlies’ wages, and one laundress’s wage and board. Kirstein said that the staff was fully occupied; no savings could be made. In addition, it was difficult to find ‘faithful and skilful’ people who were prepared to perform the unpleasant tasks involved in caring for patients suffering from plagues and the like. With regard to food, Kirstein said that no further savings were possible. Purchases and the patients’ food intake were checked daily, both by himself and by the barber surgeon. Deceased patients’ possessions were taken to the Stockholm auction house and sold to the highest bidder. The hospital then contributed to the funeral costs. Regarding the terms of the donation for the care of Catholics, Kirstein claimed that the interest on Theodor Christoffer von Antivaris’ huge donation went towards the rent for the rooms used for the care of two Catholics, one male and one female, in separate rooms. He added that even before the will, issued in 1760, the hospital would have provided care for Catholics: ‘for they are men and Swedish subjects’.^85^ However, the few Catholics who had been cared for at the hospital prior to the testament had been housed together with Lutherans.

The Austrian diplomat Theodor Christoffer von Antivaris’ will was exemplary. Others were to follow his example. Kirstein argued that, for the hospital to be sustainable without donations of estates and similar benefits, more people needed to act in the same Christian spirit as von Antivaris. This is how all foreign hospitals were established. Kirstein’s report on running costs was submitted separately. It specified the price of the food according to the order of eating: two meals a day, bread and a pint of weak beer (Sw. svagdricka) were the norm, but in addition, there were other foodstuffs and also objects such as pots, spittoons, urine glasses, linen for bandages, and so on. The records show that, in addition to the two doctors, the orderlies and the farmhands, laundry, food preparation, bread baking, the supply of various items of clothing and brewing were carried out under contract with various contractors. Expenses for about forty people hired for various tasks involved in running the institution were also noted in the report.^86^

The deputation requested certificates and reports from the management, the pharmacist and the clergyman. Wallman, the marketer, gave an account of his housekeeping in accordance with the contract he had entered into. Wallman justified his expenses, emphasised the need for an enclosure for a couple of cows so that he could ensure a supply of milk, and pointed out that he had not yet been paid in full for the years he had impeccably provided food for the sick.^87^ The laundress, Christina Boman, who carried out her work on an annual contract, also gave an account of her work and how much it cost, and the contractor Appelgren, who maintained the beds with upholstery, gave a similar account of his contract.^88^ In his book, Wolfram Kock describes several of the deputation’s many meetings. At one of them, the location of the physician and the barber-surgeon was discussed. The deputation considered that they should live at the hospital, while Acrel and Darelius argued that their private practices in the city made this impossible. This confirmed the situation that Odhelius pointed out in his speech fifteen years later. Private practice was the main source of income for both doctors. It enabled their voluntary service in the public institution. Their work was a gift to the poor and sick. However, although the physician and barber surgeon lived elsewhere, one important person did actually live in the hospital alongside the patients, orderlies, farmhands and journeymen. The upper floor of the building was namely used as a nine-room apartment for director Kirstein.^89^

The economy was central. The questions that both the medical experts and the administrators had to answer concerned exclusively the management of resources. For example, Darelius was criticised for distributing medicine at the expense of the hospital.^90^ The replies show that those who were responsible for the hospital, according to their own statements, did their utmost to purchase necessary maintenance as cheaply as possible and to minimise costs in every possible way. For example, the sale of deceased patients’ estates compensated for the cost of burial. When the deputation finally issued its report in April 1762, it proposed changes that mainly concerned the economy. In the report, the patients had been consulted, and they had all expressed their satisfaction with the care they had received. The hospital should therefore continue to exist but should not have to depend solely on donations from the public, ‘without the State itself having to contribute anything’.^91^ The deputation also proposed a contracting system for housekeeping, which in practice was already a reality. The report was discussed in Parliament, but the suggestions by the deputation were not accepted. Wolfram Kock notes: ‘The implementation of several of the deputation’s proposals – especially state appropriation – would certainly have been of great benefit to the hospital, so the rejection by the estates must be regretted’.^92^

## Conflicting interests and more hospitals

To summarise, it may be noted that the creation of the Seraphim Hospital responded to several interests. Royal involvement was admittedly limited. It mainly consisted of authorising and administering the collection of taxes. In addition to the lottery and taxes on removals, consumption, entertainment, and the flow of goods to and from Stockholm, the hospital was made possible by private funding, collections, gifts and donations. Maintenance was thus built on fragile foundations and subject to questioning. Many people contributed and wanted value for their money and labour. At the same time, some of the work was done voluntarily. Neither doctors nor managers were initially paid a salary, but later an honorarium was introduced. The economic conditions of the hospital thus partly resembled the ‘voluntary hospitals’ that emerged elsewhere in Europe in the eighteenth century, and later.^93^ The highest-ranking staff donated their labour, and private donations of varying size contributed to the maintenance and the distinction between private and public means and responsibilities was blurred. Although the hospital was considerably smaller than both Acrel and Bäck imagined, it contained rooms for the care of the sick, surgery treatments, utility rooms, and private residences for some of the staff, as well as access for the public. Its maintenance relied heavily on a variety of outsourcing solutions while keeping the lowest-ranking staff within budget.

Like its predecessors abroad, the hospital had scientific claims. The doctor and barber surgeon were responsible for the scientific work that was presented in publications, while the actual care was provided by journeymen, farmhands and orderlies. At the same time, the scientific ambitions presupposed that medical care was actually provided. However, the small number of beds shows that scientific motives were more important than satisfying the healthcare needs of the many poor people. This conclusion is also supported by the dissatisfaction of the countryside with the possibility of using the hospital and the requirement that the sick who were admitted should be curable.

The lack of financial resources was a recurring problem. The representatives of the Seraphim Hospital compared the scanty financial conditions of the hospital with those of the Danviken Hospital. Where Danviken had as income interest from neighbouring properties and could periodically be self-sufficient, the Seraphim Hospital lacked such resources.^94^ In comparison with the English and French hospitals, whose finances were based on donations from local social elites, it was clear that the donations to the Seraphim Hospital were insufficient and perhaps also the finances of the Swedish social elite. In general, the hospital appears to be an import of a foreign model for hospitals without a sufficient financial basis.

Both before the creation of the deputation and during its work, there were requests from the countryside to use the funds flowing from their parishes to the Seraphim Hospital to establish their own institutions. From Skåne, the nobility made such a request as early as 1746, but it was rejected. A renewed application for the establishment of a hospital in Lund was submitted to Parliament in 1761–2, to ‘draw the income from Skåne, Blekinge and Halland, which now goes to Stockholm’.^95^ At the beginning of the 1760s, applications were received from Härnösand, Kronoberg and Gothenburg to use the hospital funds for the counties’ own poor and sick. All requests except the one from Turku were rejected. In October 1765, however, a royal letter was issued which allowed the counties to set up hospitals at their own expense and to ‘enjoy the same rights as the hospital in Stockholm and to retain all the collections, levies and contributions that are otherwise paid annually to the hospital in question’.^96^

After 1765, hospitals were added in several towns. Hospitals in Växjö, Gävle, Lund, Nyköping, Filipstad, Karlstad, Falun, Kristianstad, Sundsvall, Kalmar, Jönköping, Västerås, Södertälje, Halmstad and Örebro were all established before 1781. Some hospitals had already been established, such as that in Mariestad, which was added in 1760 but had to close a few years later due to lack of funds, only to reopen in 1793.^97^ The County Hospitals resulted in a drastic drop in income for the Seraphim Hospital. The number of beds was halved, from forty-four to twenty-two, and admission was restricted to the inhabitants of Stockholm.^98^ The continued existence of the hospital was thus still dependent on private donations. In 1783, funds derived from customs and excise duties on tobacco were allocated to a fund for the hospital, but it was not until 1932 that the hospital became state-owned.^99^

## Conclusions

In many ways, the creation of the Seraphim Hospital followed the general pattern in Europe, but there were also differences. Medical expertise was the driving force in Sweden, not the wealthy social elite. Caring for the poor was the main argument both in the Swedish case and elsewhere, and it was certainly sincere. The establishment of the Seraphim Hospital confirms that concern for the poor, who had always been a large part of the population, was now a viable political argument. As Foucault argues, social and economic problems thus became part of the medical discourse of the time, which in the case of the Seraphim Hospital also included practical organisation, management, and the concrete budgeting of maintenance. The small number of beds, however, shows that in practice, the hospital could mainly only satisfy the scientific ambitions of the physicians, not the healthcare needs of the many poor.

A defining characteristic of eighteenth-century Europe was not only a Christian-influenced concern for the poor, but also a recognition of the poor as a potential resource. In the Swedish context, this concern was particularly focused on the needs of soldiers. The military dimension is evident in several aspects, including the fact that some of the hospital’s physicians had military backgrounds, and that the proposal for a lottery to fund the hospital was put forward by one of the many military officers among the nobility estate. Given Sweden’s involvement in numerous wars during this period (which continued until 1809), it is evident that many members of the nobility had military experience and that these officers also played a significant role in both political and civil life.^100^ The pervasive influence of the military in Swedish society highlights an important aspect of path dependency in the country’s historical development, a feature that distinguishes Sweden from several other countries.

The large number of hospitals founded in the decades after the Seraphim Hospital did not have direct medical motives, but here, the economy was an important argument; the parishes in the countryside wanted to avoid the expense of the hospital in Stockholm. The uncertainty of the hospital’s finances and the solutions adopted were based on local conditions in which the church played an important role. Both in the procurement of funds and patients, the clergyman was a key figure and very much part of the hospital organisation.

Finances were a fundamental problem for most eighteenth-century hospitals, and in Sweden, a direct reason why even rural areas wanted to establish a hospital. Local parishes wanted to keep their collections and therefore their patients. The founding of hospitals in the 1760s, therefore, brought back the medical organisation of the Middle Ages, where the church and the clergyman were crucial in providing medical care for both the body and the soul. In the case of the Seraphim Hospital, dependence on church officials deepened. Clergymen played a key role in raising financial resources, and it was clergymen who made referrals to the hospital and thus provided primary care.

Path dependency, by following the finances, went obviously to the ecclesiastical sphere. The 1760s and 1770s stand out as a time of critical junctures. The church and other influential groups protested against their collected funds going to the hospital in Stockholm. Instead of avoiding the fundraising effort, the various dioceses chose to establish hospitals themselves. In addition to concern for the morbidity of their own population, there was also a renewed threat to consider: the rapidly increasing spread of venereal diseases. To control and preferably limit venereal infections, the old ecclesiastical organisation allied itself with new medical institutions.^101^ Although the Seraphim Hospital broke with the past’s conflation of the poor and the sick by admitting only the poor with *curable* diseases, it also maintained older traditions in which the authority of the clergyman remained, and sickness and poverty were still intertwined. The challenges encountered in the establishment of hospitals in Sweden highlight the complexities involved and challenge the notion of medicalisation as a straightforward, unidirectional process.^102^

The establishment of the Seraphim Hospital and its financial situation also initiated the founding of other hospitals, which were built on similar economic foundations. Like care in medieval hospitals and shrines, medical care in the hospital was still a gift to the poor. The concepts of path dependency and critical junctures not only illuminate the influence of the past on innovation but also underscore the importance of historical research extending beyond a forward-looking focus on outcomes and consequences. Equally vital for historical understanding is the retrospective view – that is, the deepening of the historical perspective.

## Footnotes

1

Mary Lindemann , Medicine and Society in Early Modern Europe (Cambridge: Cambridge University Press 1999), 134 , 138; Guenter B. Risse , Mending Bodies, Saving Souls. A History of Hospitals (Oxford: Oxford University Press 1999), 238 ; Keir Waddington , Charity and the London Hospitals 1850-1898 (London: The Royal Historical Society 2000), 7–8 .

2

Axel C. Hüntelmann , ‘Economies of the hospital, 1790-1910’, in Axel C. Hüntelmann & O. Falk , (eds), Accounting for Health. Calculation, Paperwork, and Medicine, 1500-2000 (Manchester: Manchester University Press 2021), 109 .

3

Bronwyn Croxson , ‘The price of charity to the Middlesex Hospital, 1750-1830’, in Martin Gorsky and Sally Sheard (eds), Financing Medicine. The British Experience since 1750 (London: Routledge 2006), 24 .

4

Signild Vallgårda , Sjukhus och fattigpolitik. Et bidrag til de danske sjukhusens historia 1750-1880 (Copenhagen: Institut for social medicin, University of Copenhagen 1985); Ole Feldbaek , Anne Lokke, S L Jeppesen , Drommen om tryghed. Tusind års dansk forsikring, (Copenhagen: Gads forlag 2007), 101 .

5

Ole Georg Moseng , Ansvaret for undersåttenes helse 1603-1850, (Oslo: Universitetsforlaget 2003), 257 .

6 Guenter B. Risse, *op.cit.* (note 1), 241, 264.

7
*Svenska Akademiens Ordbok (SAOB)*: the term ‘lasarett’ refers to a hospital facility. The word ‘lasarett’ in Sweden and the word ‘hospital’ in other countries are used to describe medical facilities where people can receive care and treatment for various types of illnesses and injuries. The difference in terminology stems from historical and linguistic factors.

8

John Henderson , Pregrine Horden and Alessandro Pastore , ‘Introduction. The world of the hospital: Comparisons and continuities’, in John Henderson , Pregrine Horden and Alessandro Pastore (eds), *The Impact of Hospitals*, 300-2000 (Bern: Peter Lang 2007), 21–3.

9

Richard Wawrinsky , Sveriges lasarettsväsende förr och nu: ett stycke svensk kulturhistoria (Stockholm: author’s publisher 1906), Appendix 1.

10

Michel Foucault , The Birth of the Clinic. An Archaeology of Medical Perception (1963) (New York: Vintage Books Edition 1994), 26 ; Risse, *op. cit.* (note 1), 238.

11 Henderson *et al*, *op. cit.* (note 8), 33.

12 Fritz Dross, ‘The Invention of a Medical Institution? A Discussion of Hospitals around 1800’, *Hygiea Internationalis*, 2006, Vol. 5, Iss. 1, 92, 94, 99.

13
*Ibid.*, 99.

14

Roy Porter , ‘The gift relation: philanthropy and provincial hospitals in eighteenth-century England’, in Lindsay Granshaw and Roy Porter (eds), The Hospital in History, (London: Routledge, 1989), 149–71; Risse, *op. cit.* (note 1), 238–41.

15

Marcel Mauss , The Gift. Forms and Functions of Exchange in Archaic Societies, (London: Cohen & West LTD 1969), 10 , 37, 45; Gadi Algazi , Valentin Groebner , Bernhard Jussen (eds.), Negotiating the Gift: Premodern Figurations of Exchange, (Göttingen: Vandenhoeck & Ruprecht 2003); Johanna Thorelli , De tjänstvilliga vännernas samhälle. Abraham Brahe och den svenska eliten 1590-1630, (Göteborg: Diss. Göteborgs universitet 2023), 67–8, 74.

16

Mario Damen , ‘Princely Entries and Gift Exchange in the Burgundian Low Countries: A Crucial Link in Late Medieval Political Culture’, Journal of Medieval History, Vol. 33 (3), 2007, 233–49.

17

Geoffrey Rivett , The Development of the London Hospital System 1823-1982, (London: King Edward’s Hospital Fund for London 1986), 15 ; Waddington, *op. cit.* (note 1), 7.

18 Sandra Cavallo, ‘Charity, power and patronage in eighteenth-century Italian hospitals: the case of Turin’, in Lindsay Granshaw and Roy Porter (eds.), *op. cit.* (note 14), 93–122, Mary Lindemann, *op. cit.* (note 1), 137–8, Mathhew Thomas Sneider, ‘The treasury of the poor: hospital finance in sixteenth- and seventeenth-century bologna’, in Henderson *et al*, *op. cit.* (note 8), 98–101, Marina Garbellotti, ‘Assets of the poor, assets of the city: the management of hospital resources in Verona between the sixteenth and eighteenth centuries’, in Henderson *et al*, *op. cit.* (note 8), 119.

19 Risse, *op. cit.* (note 1), 261.

20

Tim McHugh , Hospital Politics in Seventeenth Century France. The Crown, Urban Elites and the Poor (London: Routledge 2007), 168–9.

21

Ole Georg Moseng , ‘Det forste sykehus i Nordlandene’, in Wilheln Karlsen , (ed), Medicinal-Indretningen. Nordlandssykehusets historie 1796-2020. Somatics (Oslo: Cappelen Damm Akademisk 2021), 29 .

22 Moseng, *op. cit.* (note 5), 266.

23 Risse, *op. cit.* (note 1), 4–5.

24

James Mahoney , ‘Path Dependence in Historical Sociology’, Theory & Society 29, no. 4 (2000), 50 .

25 Walter Korpi, ‘Contentious Institutions: An Augmented Rational-Action Analysis of the Origins and Path Dependency of Welfare Institutions in the Western Countries’*, Rationality and Society*, 13 (2001), 235–83.

26

Andre Sorensen , ‘Taking Path Dependence Seriously: an Historical Institutionalist Research Agenda in Planning History’, Planning Perspectives, 30:1, 2015, 22 ; John Hogan , ‘The Critical Juncture Concept´s Evolving Capacity to Explain Policy Change’, European Policy Analysis, Vol. 5, No. 2, 2019, 175 , 179.

27 Mahoney, *op. cit.* (note 24), 50.

28 McHugh, *op. cit.* (note 20), 169; Henderson et al, *op. cit.* (note 8), 32, 34.

29

Jonathan Barry and Colin Jones , ‘Introduction’, in Jonathan Barry and Colin Jones , eds, Medicine and Charity before the Welfare State, (London: Routledge 1991), 3.

30 Vallgårda, *op. cit.* (note 4), 119.

31

Karin Johannisson , Det mätbara samhället. Statistik och samhällsdröm i 1700-talets Europa, (Stockholm: Norstedts 1988), 107 : ‘Med hjälp av myriader av trägna arbetare skulle Sverige förvandlas från “kall vild öken” till ett land av myllrande välmåga’.

32 Lindemann, *op. cit.* (note 1), 121–2.

33

Otto E. A. Hjelt , Svenska och finska medicinalverkets historia 1663-1812, (Helsinki: Helsinki Centraltryckeri 1891-1893), 3 Vols, (I), 8 .

34 Wawrinsky, *op. cit.* (note 9), 15.

35 Wolfram Kock, *Kungl. Serafimerlasarettet 1752-1952. En studie i svensk sjukvårdshistoria*, (Diss. Stockholm: Karolinska institutet 1952), 23.

36 Wolfram Kock, ‘Sjukhusväsendets utveckling i Sverige’, *Svenska sjukhus. En översikt av det svenska sjukhusväsendets framväxt till 1900-talets mitt*, 3 Vols, (I), (Stockholm: Gothia 1948), 13.

37 Foucault, *op. cit.* (note 10), 34; Lindemann, *op. cit.* (note 1), 121; Jan Sundin & Sam Willner , Social Change and Health in Sweden. 250 years of Politics and Practice, (Stockholm: Swedish National Institute of Public Health 2007), 66 .

38

Roger Qvarsell , Vårdens idéhistoria. (Stockholm: Carlsson 1991); Gunnar Broberg , (ed), Til at stwdera läkedom. Tio studier i svensk medicinhistoria, (Lund: Sekel 2008); Solveig Jülich , Skuggor av sanning: tidig svensk radiologi och visuell kultur, (Linköping: Linköpings universitet 2002); Karin Johannisson , Ingemar Nilsson & Roger Qvarsell , Medicinen blir till vetenskap: Karolinska Institutet under två århundraden, (Stockholm: Karolinska Institutet University Press 2010); Anneli Drakman , När kroppen slöt sig och blev fast: varför åderlåtning, miasmateori och klimatmedicin övergavs vid 1800-talets mitt, (Diss. Uppsala: Uppsala University 2018); Karin Johannisson , Medicinens öga. Sjukdom, medicin och samhälle - historiska erfarenheter, (1990) (Stockholm: Norstedts 2013), 10–33 discusses medical history traditions; Charlotta Wolff , ‘Medicinhistoria i Finland - ett fält i föryngring’, Historisk tidskrift för Finland, 4 (2020), 411–16 gives a brief overview of current medical history.

39
*Sveriges kyrkolag af år 1686: jemte ännu gällande stadganden, genom hvilka den blifvit ändrad eller tillökt*, published by Peter Magnus Rydholm and Arvid Johan Rydén (Göteborg: D.F. Bonnier 1890).

40
*Riksarkivets beståndsöversikt.* Del 5. Centrala myndigheter och domstolar. Internationella organ. Utgiven av Bertil Johansson m. fl. Band 2. Myndigheter M-Ö. Internationella organ, (Stockholm: Riksarkivet 1999), 178.

41 Kock, *op. cit.* (note 35), 24. Danviken Hospital, located on the outskirts of Stockholm, was founded in the mid-16th century and brought together various categories of needy people.

42
*Ibid.*, 26: ‘ty kunna ock sådane försök ej bequämligare än i Lazaretter och publique sjukhus anställas’, ‘många års trägit läsande vid Academierna’.

43
*Ibid.*, 26–7.

44
*Ibid.*, 27–8.

45
*Ibid.*, 30.

46
*Ibid.*, 32.

47 Hjelt, *op. cit.* (note 30), (III), 5–11.

48 Kock, *op. cit.* (note 35), 35.

49 On the administration of the Lasarettsfonden, see Patrik Winton , ‘Krig, skulder och välfärd i Sverige 1800-1810’, Historisk tidskrift, 1 (2015), 5–34 .

50 Hjelt, *op. cit.* (note 30), (III), 12–13.

51 Kock, *op. cit.* (note 35), 32.

52 Rivett, *op. cit.* (note 17), 15; Mary Lindemann, *op. cit.* (note 1), 138; Keir Waddington, *op. cit.* (note 1), 6.

53

Barry Doyle , ‘Healthcare before Welfare States: Hospitals in Early Twentieth Century England and France’, Canadian Bulletin of Medical History, 33 (2016), 174–204 27344908 10.3138/cbmh.33.1.174. In the collected volume, *The Mixed Economy of Social Welfare. Public/Private Relations in England, Germany and the United States, the 1870s to the 1930s*, Michael B. Katz and Christoph Sachsse (eds), (Baden-Baden: Nomos Verlagsgesellschaft 1996), welfare systems in England, Germany, and the United States are examined from this perspective. The comparative analysis reveals that, although the configuration of public and private elements differed across the three countries, their interplay was fundamental in each case.

54 Kock, *op. cit.* (note 35), 425–31. The list was compiled by Gösta Råby, curator of the hospital.

55 Abraham Bäck is emphasised as one of the main driving forces behind the Seraphim Hospital, see Thomas Ihre , Abraham Bäck. Mannen som reformerade den svenska sjukvården, (Stockholm: Atlantis 2011), 210–11; Lars-Gösta Wiman , Abraham Bäck: Linnés vän och nydanare av svensk läkarutbildning, (Uppsala: Acta Universitatis Upsaliensis 2012), 9–12 .

56 Kock, *op. cit.* (note 35), 36–9. Reference is made to the original: Abraham Bäck, *Tal om Nyttan som tillflyter Läkarekonsten, af et väl inrättadt Lazaret eller Sjukhus. Hållit för Kongl. Vetenskapsacademien*, (Stockholm: Lars Salvius 1746), 26: ‘i et litet otätt krype, i fattigdom och elände, utan at någon vårdar sig rätteligen om deras mat och dryck, eller drager behörig sorg för nödvändig läkedom’.

57 Bäck, *op. cit.* (note 53), 27.

58
*Ibid.*, 34.

59 Kock, *op. cit.* (note 35), 39–42. Reference is made to the original: Olof Acrel , Genaste sätt at inrätta och underhålla et Lazaret eller Sjukhus, så at det inom få år måtte ärnå en ansenlig tillväxt. På Kongl. Vetenskapsacademiens befallning (Stockholm: Lars Salvius 1746).

60
*Ibid.*, 14: ‘som i älende förgås, i brist af tjenlig skötsel här blifva förplägade, botade och friska til deras arbete återsända’, ‘At de som i Landsorterne äro rådlösa vid svåra skadors timade kunna här angifva sig, ingå och så vida möjeligt är blifva helbregda utan vedergälning’. Given the distance to Stockholm from much of the country, this was of course an unrealistic expectation.

61 Hjelt, *op. cit.* (note 30), (I), 351–60.

62 Kock, *op. cit.* (note 35), 44–6.

63
*Ibid.*, 47.

64 Croxson, *op. cit.* (note 3), 23

65 Risse, *op. cit.* (note 1), 241, 263. The number of beds is certainly not the same as the number of patients. Despite the different measurements, it should be clear that Seraphim Hospital was very small in comparison to other hospitals.

66 Serafimerlasarettet, Medicinska kliniken I. Allmänna journaler F I a, *National Archives* (RA), Stockholm. These records are only partly preserved.

67 Olof Acrel, *Chirurgiske händelser, anmärkte uti kongl. Lazarettet, samlade och genom utdrag af dagboken til printcket befordrade på höglofloflige kongl. Lazarets directionens befallning*, (Stockholm: Lor. Ludv. Grefing 1759), contains a selection of the patients. According to the preface, only the rarest cases are described. An expanded edition, published in 1775, also describes procedures and patients he treated during his stay abroad in the French army, and this volume also includes case descriptions by others. This material is very extensive and is left aside here.

68 Kock, *op. cit.* (note 35), 51–61. These discussions cannot be dealt with here for reasons of space.

69
*Ibid.*, 135, reproduces the 1755 regulations in facsimile. In 49 paragraphs the internal life of the hospital was regulated.

70
*Ibid.*, § 42: ‘emot de Sjuka skola wisa all höflighet och ömhet, samt dem aldrig med hårda ord bemöta’.

71
*Ibid.*, § 40: ‘Denna Betjening, bör ständigt uti deras rum tilstädes wara, at om något av de Sjuka fordras dem tilhanda gå’.

72 For example, on 1761-02-19, *Inrikes Tidningar* published the annual report for 1760, when the hospital treated 348 patients.

73 Kock, *op. cit.* (note 35), 175.

74 Abraham Bäck, Underdånigst memorial den 23 November 1752, Kort 1157, Autografsamlingen, *Royal Library* (KB), Stockholm.

75

Joh. Lor. Odhelius , K. Lazarettet i Stockholm, beskrifvit uti et Tal, inför Kongl. Vetenskaps Academien, Vid praesidii nedläggande den 2 Nov 1776, (Stockholm: Johan Georg Lange 1776), 6 : ‘15 eländige uslingar’.

76
*Ibid.*, 21–2: ‘Vårt folks allmänna lynne, som aldrig söker hjelp förr, än yttersta nöden kräfver, utan lider hällre alt, i det längsta, samt låter Qvacksalfvare bedraga sig ända till kläderne’.

77
*Ibid.*, 22: ‘inkastas ofta döende personer på gården, hvilka då måste intagas, och endast öka död-listan samt begrafningskostnaden. Det har ock hänt, at verkligen döde personer blifvit ditförde och afkastade’.

78
*Ibid.*, 39.

79
*Ibid.*, 37.

80 Kock, *op. cit.* (note 35), 67.

81 Frihetstidens utskottshandlingar, Lasarettsdeputationen, vol. 1, *National Archives* (RA).

82
*Ibid.*, 213–14.

83
*Ibid.*, 217: ‘[…] utsprida bevisliga osanningar bland det allmänna. Jag anser det emedlertid med förakt och är säker om bättre vitsord af dem som dyrckar billighet och sanning’. Acrel didn’t explain what kind of experiments he was referring to.

84
*Ibid.*, 222.

85
*Ibid.*, 235: ‘ty de äro manniskor och swenska undersåtare’.

86
*Ibid.*, 242–4.

87
*Ibid.*, 253–4, 256–7.

88
*Ibid.*, 271, 273–4.

89 Kock, *op. cit.* (note 35), 48.

90
*Ibid.*, 70.

91
*Ibid.*, 71: ‘utan att sjelfwa Staten måtte något bidraga’

92
*Ibid.*, 71: ‘Genomförandet av åtskilliga av deputationens förslag – speciellt statsanslaget – hade säkert varit av stor nytta för lasarettet, varför ständernas avslag måste beklagas’.

93 Rivett, *op. cit.* (note 17), 15; Waddington, *op. cit.* (note 1), 63; Risse, *op. cit.* (note 1), 6, 238–9; Martin Gorsky and Sally Sheard, ‘Introduction’, in Martin Gorsky and Sally Sheard, eds, *Financing Medicine. The British Experience since 1750*, (London: Routledge, 2006), 3.

94 Hüntelmann, *op. cit.* (note 2), 109–12 shows how the Charité outside Berlin was self-sufficient at the turn of the century 1800; Eva Eggeby, *Vandringsman*, *här ser du en avmålning av världen. Vårdande, vård och ekonomi på Danvikens dårhus 1750-1861*, (Diss. Stockholm: Almqvist & Wiksell International 1996), 118–19 shows that equity grew in importance over time.

95 Hjelt, *op. cit.* (note 30), 1891-1893, (III), 32: ‘draga inkomsterna ifrån Skåne, Blekinge och Halland, som nu gå till Stockholm’.

96
*Ibid.*, 35: ‘få nyttja samma rättighet, som lazarettet i Stockholm och behålla alla de insamlingar, collecter och sammanskott, som eljest till berörde lazarett årligen utgå’.

97 Wawrinsky, *op. cit.* (note 9), appendix 1.

98 Kock, *op. cit.* (note 35), 72.

99
*Ibid.*, 75.

100

Gunnar Artéus , Krigsmakt och samhälle i frihetstidens Sverige, (Stockholm: Militärhistoriska Förlaget 1982), 49, 386 .

101

Nils Thyresson , Från Fransoser till Aids. Kapitel ur de veneriska sjukdomarnas historia i Sverige, (Stockholm: Carlssons 1991), 41–2; Anna Lundberg , Läkarnas blanka vapen. Svensk smittskyddslagstiftning i historiskt perspektiv, (Lund: Nordic Academic press 2008), 42–4; Agneta Rahikainen , Smittans rike . Om syfilis i konst, kultur och kropp, (Stockholm: Natur & Kultur 2021), 56 .

102 Henderson et al, *op. cit.*, (note 8), 33.

